# Phosphorous Nutritional Level, Carbohydrate Reserves and Flower Quality in Olives

**DOI:** 10.1371/journal.pone.0167591

**Published:** 2016-12-01

**Authors:** Ran Erel, Uri Yermiyahu, Hagai Yasuor, Dan Cohen Chamus, Amnon Schwartz, Alon Ben-Gal, Arnon Dag

**Affiliations:** 1 Gilat Research Center, Agricultural Research Organization of Israel, Israel; 2 Institute of Plant Sciences and Genetics in Agriculture, The Robert H. Smith Faculty of Agriculture, Food and Environment, The Hebrew University of Jerusalem, Rehovot, Israel; Institute of Genetics and Developmental Biology Chinese Academy of Sciences, CHINA

## Abstract

The olive tree is generally characterized by relatively low final fruit set consequential to a significant rate of undeveloped pistils, pistil abortion, and flower and fruitlet abscission. These processes are acknowledged to be governed by competition for resources between the developing vegetative and reproductive organs. To study the role of phosphorus (P) nutritional level on reproductive development, trees were grown under four levels of P for three years in large containers. Phosphorus nutritional level was positively related to rate of reproductive bud break, inflorescence weight, rate of hermaphrodite flowers, pistil weight, fruitlet persistence, fruit set and the consequential total number of fruits. The positive impact of P nutrition on the productivity parameters was not related to carbohydrate reserves or to carbohydrate transport to the developing inflorescence. Phosphorous deficient trees showed significant impairment of assimilation rate, and yet, carbohydrates were accumulated in inflorescences at levels comparable to or higher than trees receiving high P. In contrast to female reproductive organs, pollen viability was consistently higher in P deficient trees, possibly due to the enhanced carbohydrate availability. Overall, the positive effect of P on female reproductive development was found to be independent of the total carbohydrate availability. Hence, P is speculated to have a direct influence on reproductive processes.

## Introduction

Olive trees (*Olea europaea* L.) produce a large number of flowers, only a small portion of which eventually set fruits [1]. As a result of either nutrient or assimilates competition among the developing fruitlets, a massive abscission of fruitlets occurs soon after fertilization. Thus, final fruit set is determined roughly 5–6 week after anthesis [2, 3]. Fruit set is greatly dependent on environmental conditions, bearing history [4–6] and genetic traits [7]. To reach economically sufficient yields, in addition to a satisfactory number of flowers, flower quality is requisite. Flower quality is determined by multiple characteristics which elevate the probability of successful fertilization and fruitlet persistence [8, 9]. In the olive, flower characteristics are acknowledged to play a role in fruit setting and fruit development including: rate of pistil abortion, embryo-sac development, stigma receptivity, ovule longevity, ovary size, carbohydrate content, pollen viability and nitrogen (N) content [4, 10].

The olive is an andromonoecious species, bearing a mixture of perfect (hermaphrodite) and male (staminate) flowers with aborted (undeveloped) pistils. Pistil abortion is a mechanism of resource management responding to demand, availability and environmental conditions [11]. Enhanced competition or depleted resources elevates the extent of pistil abortion, thus simultaneously reducing fruiting potential and preserving carbon investment in flowers [12]. Within the perfect flowers, larger ovary size is considered a positive trait, closely related to sink strength [13]. Large ovaries are generally associated with superior probability to set fruit [12]. At flowering, massive amounts of pollen are released from the anthers to the air [14], a typical feature of wind-pollinated taxon. Of the vast numbers of grain released, only a portion is viable, can germinate on the stigma and fertilize one of the four ovules. Pollen viability is largely a genetic trait [15, 16] shown to be similar for staminate and perfect flowers [12]. The olive is a partly self-incompatible species for which cross-pollination results in higher fertilization levels [1, 17]. While requiring a large quantity of viable airborne pollen [16, 18, 19], cross-pollination is rarely the limiting factor for fruit set in traditional cultivation regions [20].

In general, flower carbohydrate reserves support pollen tube growth and early development of embryos [21] and therefore are thought to be significant enhancers of productivity [22–24]. In olive, flowering induction does not seem to rely on carbohydrate reserves as shown by Stutte and Martin [25], using manipulation of light and CO_2_, and by Cherbiy-Hoffmann et al. (2015), using manipulations of light intensity. In a field trial, Bustan et al. [26] found no major effect of fruit load on total carbohydrate reserves and thus concluded that the role of the reserves mainly involves tree survival. Regarding olive flower development, Reale et al. [27] reported a close correlation between starch content and hermaphrodite flower formation. Additionally, indirect evidence of the centrality of carbohydrate availability to productivity was found in girdling trials. Girdling, which elevates shoot carbohydrate availability, was shown to increase the percentage of perfect flowers, fruit set and productivity [28, 29].

As stated above, flower quality characteristics are affected by genetic and environmental factors. Information regarding the interaction between flower quality, productivity and mineral nutrition is limited solely to N [30, 31]. Recent evidence indicates a positive contribution of phosphorous (P) on olive productivity [32]. Phosphorus is an essential macro-element for numerous plant functions including carbohydrate metabolism [33] and transport [34]. In spite its relative abundance, deficient P frequently limits plant production in many arable soils [35]. This is mainly due to restricted soil P mobility and availability, especially when soil moisture is low [36]. Irrigation has been reported to improve P uptake in olives [37].

Olive trees are capable of bearing fruits in poor soils and harsh environments, hence, the crop was traditionally grown under extensive rain-fed culture associated with low productivity [38]. Due to this, and also probably a result of the tree's extensive root system and symbiosis with mycorrhizal fungi [39], P deficiencies in olive are considered rare, and P fertilization is not typically recommended [40]. Over the past three decades, intensive, high density, irrigated orchards have become common. As a part of modern production systems, irrigation has been credited with substantially increasing olive productivity [41–44]. Due to high rates of growth and production and the unique techniques for fertilizer application occurring in modern plantations, olive tree response to nutrients and fertilizer requirements are expected to be effected. However, the role of fertilization in olive productivity in general, and the role of P in productivity of intensive orchards specifically, have not been thoroughly investigated.

In a previous study, we found a continuous increase in fruit set in response to increased P application rate in young olive trees [45]. Further investigation confirmed this observation and revealed strong association between P nutritional status, rate of perfect flowers and fruit set [32]. Although the positive effect of P on olive production is well established, the mechanisms in which P nutrition contributes to olive productivity are not yet understood. Phosphorus is acknowledged as essential for assimilate up-load and transport and thus may restrain sink competition [34]. Successful fruit set often relies on efficient transport of assimilates to fertilized flowers rather than building of large starch reserves. We hypothesized that, under intensive cultivation, improved P nutrition enhances carbohydrate availability. Hence, higher productivity in response to increased P may be a result of diminished competition on assimilates and nutrients during flowering and fruitlet development. The objective of the current study was to investigate the effect of P nutrition on carbohydrate dynamics in developing flowers, on flower quality, and on productivity in irrigated olives.

## Materials and Methods

Two-year old ‘Barnea’ olive trees were planted in March 2011 at the Gilat Research Center, Israel (lat. 31°20_N, long. 34°39_E). The trees were grown in 150 l containers filled with type 2 (0–2 mm) granular perlite (Agrekal, Habonim Industries Ltd, Israel). The experiment was arranged in a randomized block design with 10 replicates. Initially, the trees were irrigated excessively and fertilized with liquid commercial fertilizer 7-3-7+micro elements (Fertilizers and Chemicals ltd, Israel). The irrigation solution contained 4.51, 0.84 and 3.74 mM of N, P and potassium (K), respectively. Differential nutrient application treatments were initiated on 16 May 2011. Treatment P mineral concentration and nomenclature are given in Table 1. The remaining nutrients were applied to all trees at identical levels. Target nutrient concentration was achieved by proportionally dissolving salts of: KH_2_PO_4_, KNO_3_, NaNO_3_, MgSO_4_, and NH_4_NO_3_. Micro-nutrients were supplied as EDTA chelate solution (Fertilizers and Chemicals Ltd, Israel) with final solution concentrations of: 4.71mM NO_3_; 0.49 mM NH_4_; 2.85 mM K; 0.84mM Ca; 0.56 mM Mg; 13.8 μM Fe, 5.5 μM Mn, 3.9 μM Zn and 0.4 μM Cu. The irrigation solution pH and EC were 7.1±0.5 and 1.0±0.1 dS m^-1^, respectively. Trees were irrigated daily in excesses via an automatic drip irrigation system. Irrigation solutions for each treatment were supplied from 1.5 m^3^ water tanks containing. Irrigation water pH, EC, mineral concentrations and drainage volume were monitored twice a month to ensure treatment accuracy. Following the first harvest, trees were pruned to allow sufficient light penetration.

**Table 1:** Averages ± standard deviation of: phosphorus (P) concentration in irrigation solution, and corresponding P leaf concentration measured during the flowering periods, percentage of buds evolved into inflorescences, flowers to fruits, and total number of fruit per tree, all for each of the two years of study. Different letters indicate values with significant differences at *p*<0.05 (6 repetition).

	Solution P (mg l^-1^)	Leaf P (g 100g^-1^)		Reproductive bud break (%)				Flowers per inflorescence				Perfect flowers (%)				Fruit set (%)				Fruit tree^-1^			
		May-12	May-13	2012		2013		2012		2013		2012		2013		2012		2013		2012		2013	
**P1**	0.1 ± 0.1	0.04 ± 0.00	No data	1.5	C	No data		13.5	C	No data		45.3	B	No data		2.6	B	No data		2	B	No data	
**P2**	1.0 ± 0.1	0.06 ± 0.00	0.06 ± 0.01	11.8	B	10.4	A	18.6	B	17.6	B	51.4	B	88.8	B	3.5	B	1.6	B	360	B	695	B
**P3**	10.3 ± 0.5	0.13 ± 0.02	0.12 ± 0.01	33.9	A	15.3	A	19.9	AB	20.3	A	77.4	A	90.4	AB	8.0	A	4.4	A	5,563	A	2,776	A
**P4**	26.3 ± 0.9	0.16 ± 0.03	0.16 ± 0.02	32.5	A	12.7	A	20.7	A	20.2	A	81.6	A	93.3	A	8.2	A	4.3	A	4,889	A	2,718	A

### Measurements

#### Biomass

On May 2012, subsequent to fruit set, four trees per treatment were cut dawn and divided to four fraction: leaves, branches, limbs and trunk, each was weighted and representative sample was mulched, weighted and oven dried to determine water content. The same procedure was determined on the remaining trees at the end of the trial (July 2013).

#### Mineral analysis

In May 2012 and 2013, shortly after blooming, young fully expanded leaves were sampled according to the procedure described by Beutel et al. [46]. Total P concentration in the leaves was determined after digestion with sulfuric acid and peroxide. Concentration of P was determined with an autoanalyszer (Lachat Instrument, Milwaukee, Wis., US).

#### Flowering parameters

On 2012, trees were divided into two groups: four trees per P level were used for frequent destructive inflorescence sampling and the remaining six trees were used for determination of additional parameters. On 2013 the data was collected from the remaining six trees (frequent inflorescence sampling was not preformed). Detailed description of data collection methods was given in a previous study (Erel et al.[32]. In brief, rate of reproductive bud break was determined by counting the number of inflorescences on 10 denoted branches per tree (n = 6) divided by the number of leaves. Number of flowers and number of perfect flowers were counted on 10 selected inflorescences per tree having a mixture of opened and close flowers. In order to determine fruit set percentage, in March of each year flowers were counted on six marked branches per tree. The branches used for fruit set determination were selected at the perimeter of each tree, at 1.5–2.0 meter height. In June, the number of fruits on the marked branches was counted and fruit set was defined as the ratio of fruits to flowers per branch. In July each year all fruits were manually removed and counted. During flowering, the date of full bloom was determined for each tree by visual assessment. Full bloom (FB) was defined according to Rapoport and Rallo [2], as the day on which more than 50% of flowers were open on more than 50% of the branches.

#### Inflorescence weight

After inflorescences appeared, 20 inflorescences per tree (n = 4) were sampled weekly, until petals dropped. To avoid interfering with productivity parameters, only four repetitions were used for the frequent inflorescence sampling, these trees were not used for fruit-set and fruiting determination. Sampled inflorescences were later used for carbohydrate analysis. A number of sampling points of P1 are missing due to the low flowering intensity which produced insufficient numbers of inflorescences for frequent destructive analysis.

#### Gas-exchange

Net CO_2_ assimilation rate (*A*_*n*_) and stomatal conductance of H_2_O vapor (*g*_*s*_) were measured simultaneously by a portable gas exchange measuring system (LI-6400-40 LI-COR, Inc., Lincoln, NE). Young, developed and fully illuminated leaves were measured around each tree’s perimeter. Chamber conditions were set to environmental CO_2_ concentration of 400 μmol CO_2_ mol^-1^, photosynthetic photon flux density of 1,000 μmol mol m^-2^ s^-1^, humidity of 15 mmol H_2_O mol^-1^ and block temperature of 25°C. Measurements were taken on clear days on the 7^th^, 10^th^ and 14^th^ of May 2012. Four measurements per tree and four trees per treatment were used (n = 16).

#### Pistil weights

At FB recently opened inflorescences were removed from four trees per treatment and immediately brought to the lab. Petals were discarded and pistils were gently removed from the sepals by pressing their base using tweezers. Pistils were determined to be apical or basal according to their position on the inflorescence. For each tree, at least 100 pistils were weighed.

#### Dynamic of pistil abscission

Using the method adopted from Rapoport and Rallo [2], at FB, four branches per tree from six trees per treatment were denoted and flowers were counted. Throughout May, these branches were shaken into bags daily. The abscised pistils found in the bags were divided into perfect or imperfect classes and counted. Data are presented as relative abscission rate.

#### Pollen viability

Two methods were used to estimate pollen viability. In 2012 Alexander’s reagent [47] was used to stain pollen at FB from eight inflorescences per treatment. Some 200 grains per inflorescence were examined. Only purple stained grains were considered potentially viable. In 2013 both Alexander’s reagent and fluorescein diacetate (FDA) staining methods were applied. FDA staining was performed as described by Pinney and Polito [48].

#### Starch and soluble carbohydrates (SCH)

Starch concentration in inflorescences was determined using the same samples used to assess inflorescence weight described above. Leaves and flowers were frozen in liquid nitrogen, lyophilized, milled and stored at -20°C until analysis. Soluble carbohydrates (SCH) were extracted three times with 80% ethanol at 80°C. Starch was determined in the residual by digesting ethanol insoluble residue with amyloglucosidase as described in Vishnevetsky et al. [49]. Glucose in solution was determined by colorimetric reaction with dinitrosalicylic acid according to Miller [50]. SCH in the developed inflorescences were determined using the ethanol extracts described above. The extracts were evaporated to dryness and re-dissolved in 4 ml double distilled water from which aliquots of 500 μl were lyophilized. The dried material was derivatized for 90 min at 37°C (in 40 μl of 20 mg ml^−1^ methoxyamine hydrochloride in pyridine), followed by a 30 min treatment with 70 μl of N-methyl-N(trimethylsilyl) trifluoroacetamide at 37°C and centrifugation. After derivatization, the SCH were analyzed via Agilent 6850 GC/5795C MS (Agilent Technology, Palo Alto, CA). One μl was injected in splitless mode at 230°C. Helium carrier gas was used at flow rate of 1 ml min^-1^. Chromatography was performed using a HP-5MS capillary column (30 m × 0.250 mm × 0.25 μm). Mass spectra were collected at 2.4 scans sec^-1^ with an m/z 50–550scan. MS temperature was set to 230°C. Both ion chromatograms and mass spectra were evaluated using MSD ChemStation E.02.00.493 software. Carbohydrates were identified by comparison of retention time and mass spectra with those of authentic standards (Sigma-Aldrich, Israel) and each SCH was quantified using a calibration curve.

### Statistical analysis

Data were analyzed using JMP 10.0 (SAS Institute). The four P levels can be tested by regression as continuous independent variable, however, three to four P levels are hardly sufficient for regression analysis. We choose to refer P level as discrete variable (i.e. very high, high, low and deficient) and to use ANOVA (qualitative). Effect of treatments was analyzed using one-way ANOVA (Tukey-Kramer multiple comparison test).

## Results

The three experimental years allowed two successive fruiting seasons: 2012 and 2013. Subsequent to the first fruiting year (two years after treatment initiation), the low P treatment (P1, 0.1 mg l^-1^ P) exhibited severe deficiency symptoms including essentially zero tree growth or production (data not shown). Therefore, P1 was discarded and not analyzed in the second season.

### Biomass

Trees biomass was limited by P only when leaf P dropped below 0.1% (i.e. treatments P1 and P2), while at the high P levels biomass accumulation was not affected. At flowering (May 2012) average shoot dry weight was 0.7, 4.0, 10.0 and 10.3 kg tree^-1^ for P1–P4 respectively. Average shoot biomass by the end of the experiment (July 2013) was 11.3, 22.6 and 23.6 kg tree^-1^ for P2–P4 respectively. Generally, the weight leaves, branches limbs and trunk was closely related to the total biomass and thus, is not presented.

### Nutrient concentration in leaves, reproductive buds, fruit set and fruit number

In Table 1, P levels in irrigation solution and the resulting leaf P, rate of reproductive buds, flowers per inflorescence, perfect flower ratio, fruit set and fruit number per tree are presented. One year after treatment initiation, P foliar concentration was substantially affected by application level. The highest P treatment, P4, had leaf P concentration of 0.16%, four times greater than the lowest treatment (P1, 0.04%). Comparable levels of leaf P were found in the second season, in May 2013. The two successive fruiting years of the study differed greatly regarding winter-spring bud break rate and thus, in fruiting potential. Such a seasonal effect is common in the olive and may be stemming from inhibitory effect of 2012 fruit-load, depleted resources or dissimilar winter temperatures. In 2012, reproductive bud-break percentage was affected by P availability, increasing from 1.5% in P1 up to 32–34% at the two highest P levels P3 and P4. In the following season, bud-break was noticeably lower (10–15%) and no significant differences were found between the three remaining P levels. The number of flowers per inflorescence increased with P level, yet the two highest P levels were not statistically different. The ratio of perfect flowers continuously increased with P level and was highest in at the highest P level in both seasons. For any P level, the ratio of perfect flowers was higher in 2013, the season with lower flowering intensity. Fruit set was relatively high in 2012 and a significant effect of P level was found both years. In 2012, fruit set ranged between 2.6 to 8.2% for P1 and P4, respectively and in 2013 from 1.6 to 4.3% for P2 and P3, respectively. The combined effect of flowering level and fruit set resulted in a strong effect of P treatment on fruit number per tree; ranging from 2 to 4,889 for P1 and P4, respectively, in 2012, and from 695 to 2,718 for P2 and P4, respectively, in 2013. The trees receiving the two highest P levels, P3 and P4, had similar number of fruit.

### Inflorescence development

**Fig 1:**
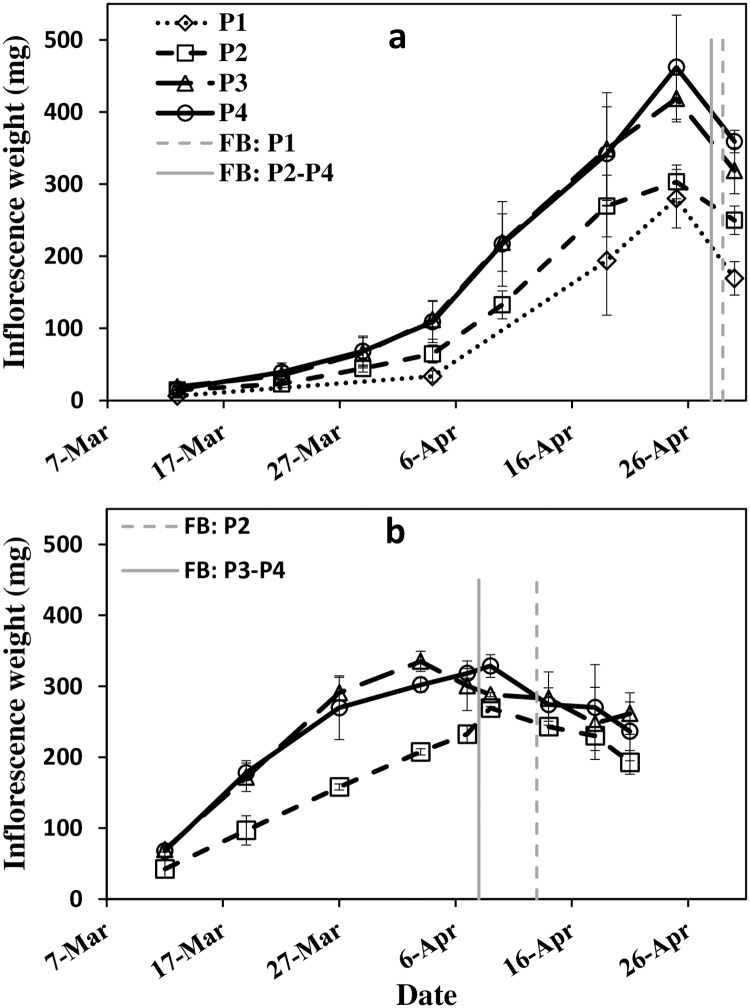
Average inflorescence fresh weight during flower development for olive trees receiving P treatments (0.1, 1, 10 and 26 mg l^-1^ P in irrigation for P1–P4 respectively, n = 4). 2012 (a) and 2013 (b). Error bars are standard deviation. Vertical lines denote the date of full bloom (FB) by visual evaluation.

Phosphorus affected inflorescence weight from early developmental stage, at the beginning of March, throughout the inflorescences' developing period. Higher inflorescence weight was evident with increasing P, up to P3 (Fig 1). The retarded development of the low P treatment was reflected in a slight delay in timing of FB. During both seasons, and regardless of P level, inflorescence fresh weight started to decline slightly before FB. In 2012, for any given P level, maximum inflorescence weight was remarkably higher and FB was delayed compared to 2013.

### CO_2_ assimilation and stomatal conductance

**Fig 2:**
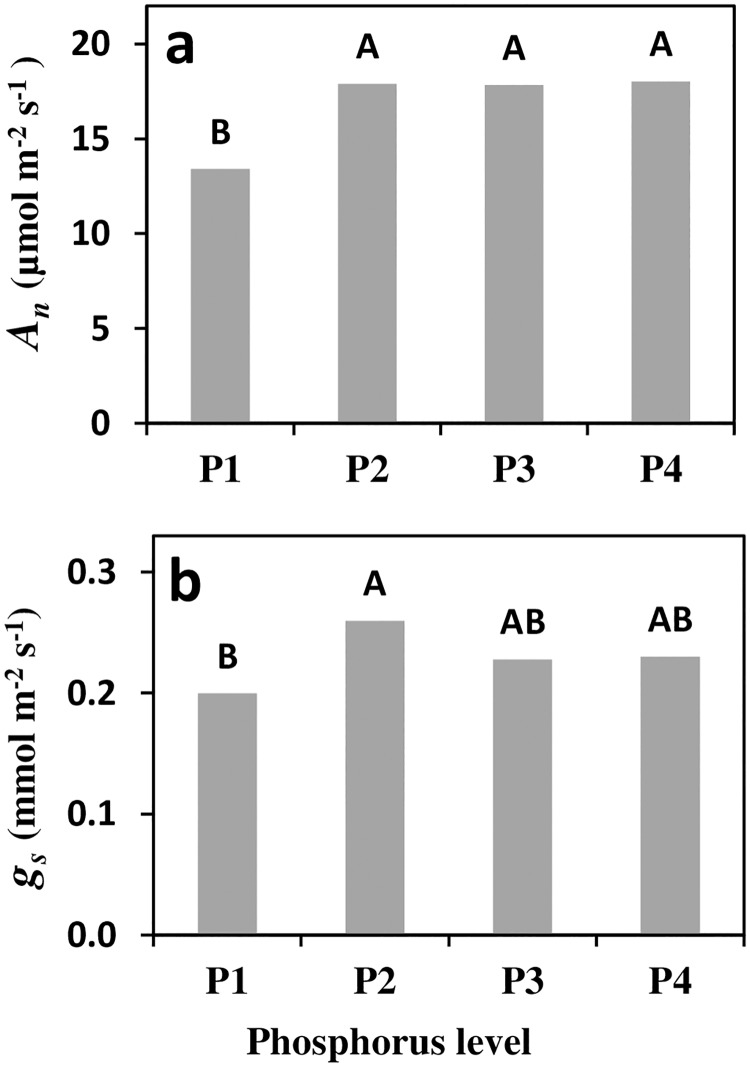
Assimilation rate (a, *A*_*n*_) and stomatal conductance (b, *g*_*s*_) of olive trees in response to four phosphorus (P) levels (0.1, 1, 10 and 26 mg l^-1^ P in irrigation for P1–P4 respectively). Measurements were taken between 10:00–12:00 over three days in early May 2012. On each day, four trees per treatment were measured (n = 16). Different letters indicate significant differences at *p*<0.05.

Net assimilation rate (*A*_*n*_) was significantly lower only at the lowest P level. P1 had average *A*_*n*_ of 13.4 μmol m^-2^ s^-1^ while P2–P4 all had 25% higher *A*_*n*_ values in the range of 17.8–18.0 μmol m^-2^ s^-1^ (Fig 2). Stomatal conductance (*g*_*s*_) was also found to be lowest at P1, but the difference was less pronounced and was significantly lower only compared to treatment P2. Relative to P2–P4, the average reduction in *g*_*s*_ of P1 trees was 16%.

### Starch content and soluble carbohydrates in developing inflorescences

**Fig 3:**
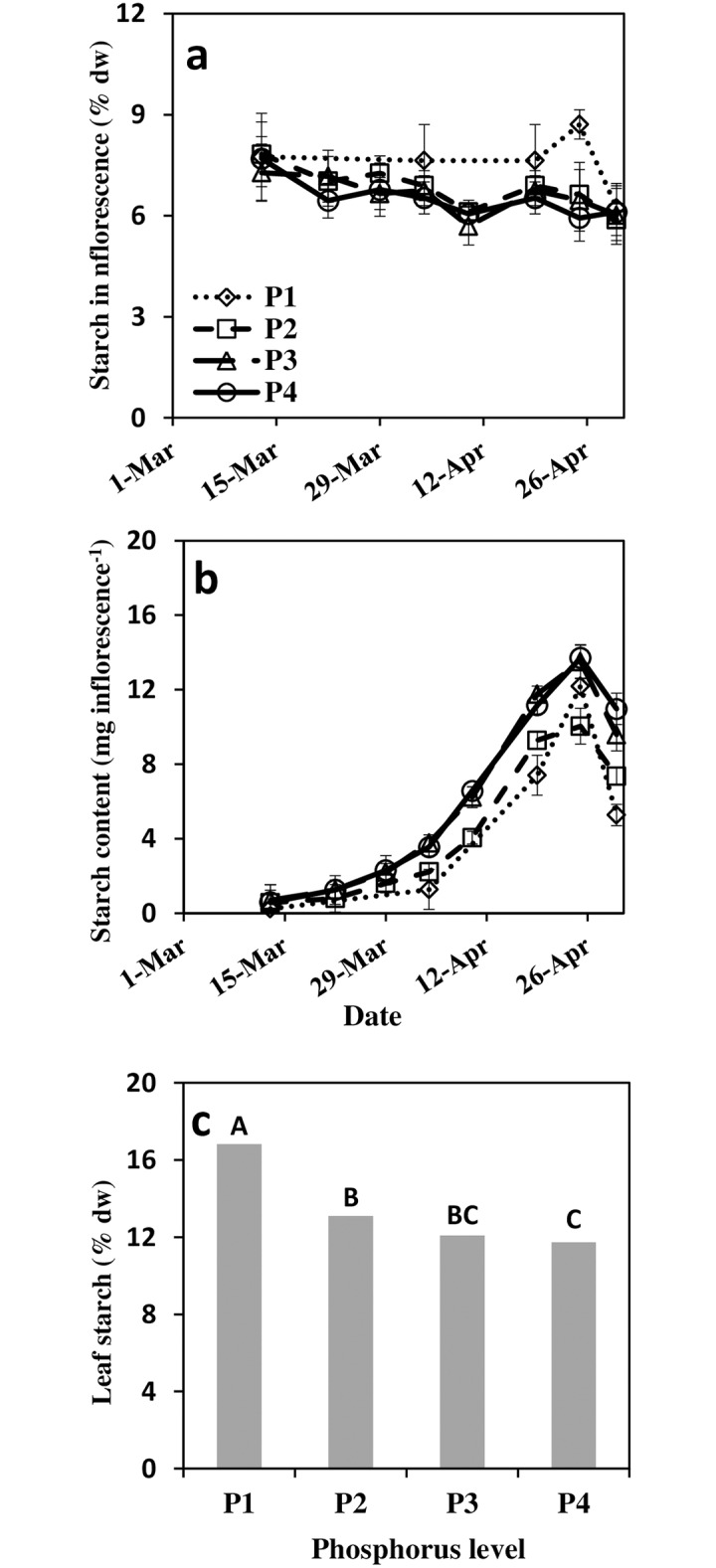
Starch concentration (a) and starch content (b) in developing inflorescences and starch concentration in leaves (c) as a function of four phosphorus levels (0.1, 1, 10 and 26 mg l^-1^ P in irrigation for P1–P4 respectively). For the inflorescences, each point represents average of four trees per treatment and two repetitions per tree (n = 8). Error bars indicate standard deviation. Leaves were sampled on May 18^th^ 2012. Each point represents the average of three trees per treatment and eight repetitions per tree (n = 24), different letters indicate significant differences at *p*<0.05.

**Fig 4:**
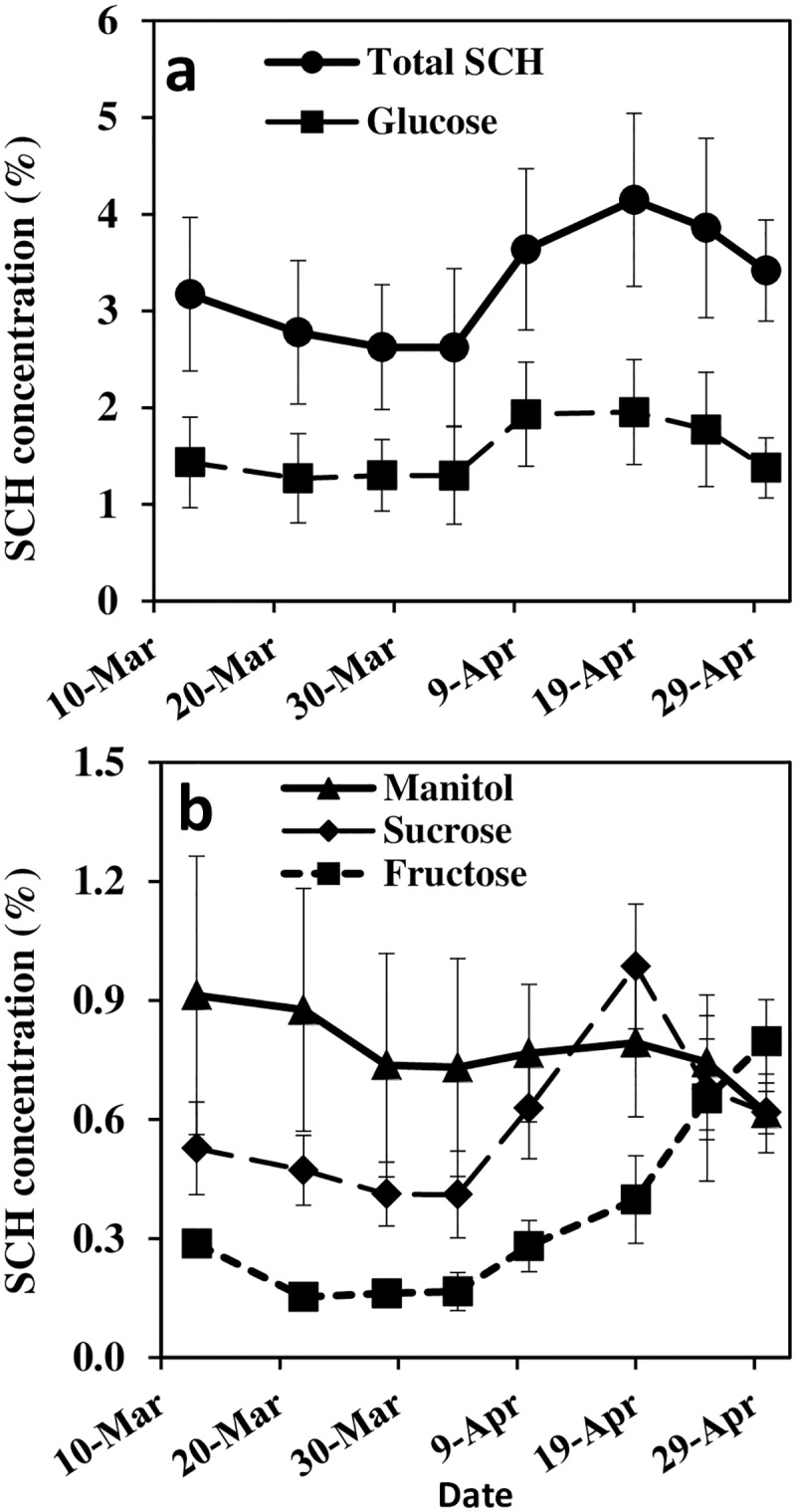
Average concentrations of total soluble carbohydrates (SCH) and glucose (a) and mannitol, sucrose and fructose (b) in the developing inflorescences from emergence to full bloom (March-April 2012). Since phosphorus level had no effect on SCH all measurements were evaluated together (n = 16). Error bars indicate standard deviation.

Starch content (dry weight basis) in the inflorescences at the earliest stage of development was highest, roughly 7.7% and gradually decreased with inflorescence development to ~ 6.0% shortly after FB (Fig 3a). Treatments P2–P4 were comparable while P1 had consistently higher starch concentration at all except the first and last sampling dates. Starch content per inflorescence (multiplying concentration by biomass) accumulated at a relatively low rate from initiation till the end of March and then accumulated at a higher rate until FB (Fig 3b). Leaf starch concentration was determined about three weeks after FB. Similar to that in the inflorescence, P1 had the highest starch concentration, 16.8%, significantly higher than treatments P2–P4 which had leaf concentrations of 13.1–11.7% (Fig 3c). Unlike starch, SCH were not affected by P level and thus the average of the four treatments is presented together (Fig 4). In general, the most abundant SCH in the inflorescence was glucose followed by mannitol, sucrose and fructose, the last three SCHs had comparable concentrations at FB. Myo-inositol was identified in very low concentrations in all treatments (data not shown). The total concentration of SCH tend to decrease from early March to early April and then sharply increased, parallel to the sharp increase in inflorescence growth rate (Fig 1). During April, SCH was high and subsequent to anthesis, SCH decreased again. The various SCHs generally followed the described pattern with minor fluctuations. Somewhat exceptional was the fructose concentration which continuously increased with inflorescence development.

### Pistil weight

**Fig 5:**
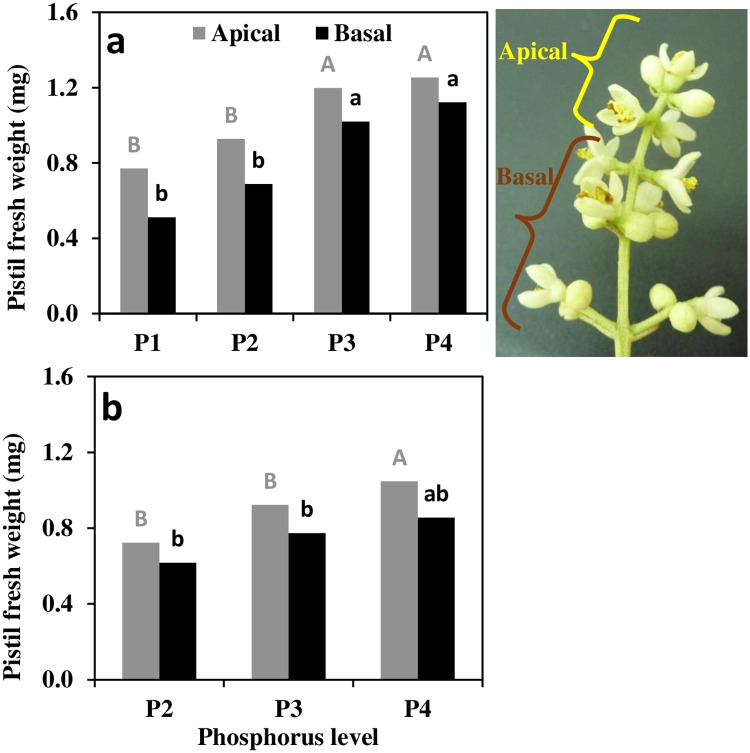
Pistil fresh weight as affected by P level. Measurements were taken proximate to full bloom, 30^th^ April 2012 (a) and 7^th^ May 2013 (b) as function of P level (0.1, 1, 10 and 26 mg l^-1^ P in irrigation for P1–P4 respectively). Different letters indicate significant differences between treatments at *p*<0.05.

Generally, pistils in the apical position had greater mass than in the basal position (Fig 5). The difference in weight between basal and apical positions was very pronounced under low P, with 34% more mass in the apical position of P1 inflorescences compare to merely 11% more in P4. Gradual increase in pistil weight with increased P availability was observed in both pistil positions; from 0.77 g in P1 to 1.25 mg in P4 for the apical position and from 0.51 in P1 to 1.12 g in P4 for the basal position. In 2013, a comparable trend, with absolute values consistently lower than in 2012, was observed.

### Abscission dynamics of male and perfect flowers

**Fig 6:**
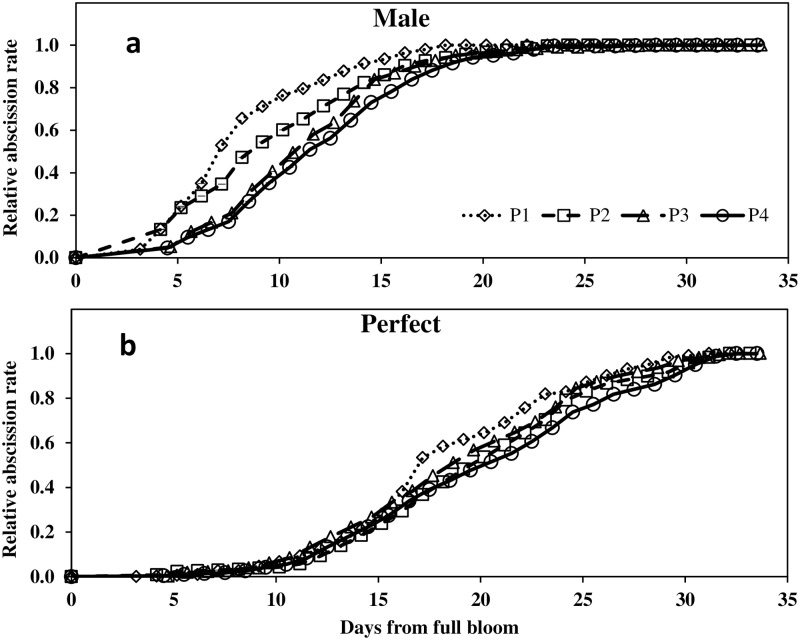
Relative abscission dynamics of male (a) and hermaphrodite (b) pistils as a function of phosphorus level (0.1, 1, 10 and 26 mg l^-1^ P in irrigation for P1–P4 respectively). Each point represents the average of four trees per treatment and five flowering branches per tree (n = 20).

The dynamics of abscission were determined only in 2012. The data was normalized according to the date of FB of each individual tree. Regardless of P level, male flower abscission started four days after FB and nearly all male flowers abscised by 20 days after FB. Perfect flowers persisted much longer and abscission started about 10 and continued until 30 days from FB. Male flowers of the two low P treatments tended to abscise earlier than those of P3 and P4. For example; 50% of male flower abscission occurred five days earlier in P1 compare to the high P treatment (Fig 6). Perfect flowers remained longer on the inflorescence and the effect of P level was less pronounced. In spite of this, P1 exhibited slightly accelerated abscission between 17 and 24 days after FB.

### Pollen viability

**Fig 7:**
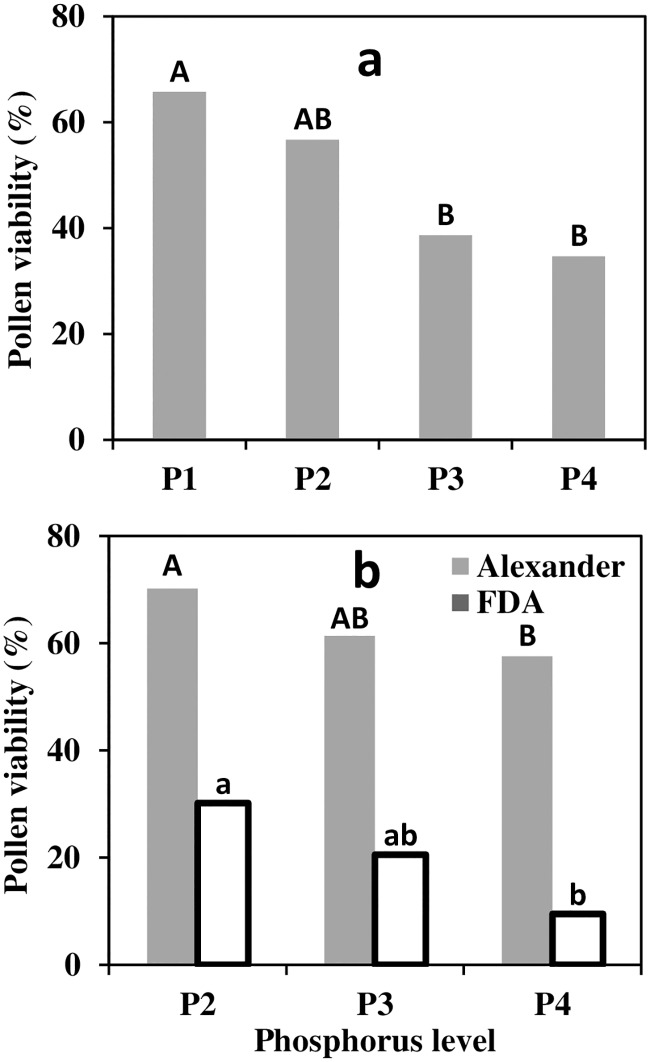
Pollen viability as affected by phosphorus levels (0.1, 1, 10 and 26 mg l^-1^ P in irrigation for P1–P4 respectively). In 2012 (a) measured by Alexander reagent and in 2013 (b) by both Alexander reagent and fluorescein di-acetate (FDA) method (200–300 flowers per treatment). Different letters indicate significantly differences at p<0.05.

Pollen viability was negatively affected by P availability. The pollen viability, as measured by Alexander’s reagent, ranged from 66% in P1 to 35% in P4 in 2012, and from 70% in P2 to 58% in P4 in 2013. The FDA method indicated similar trend with lower values, 30% to 9.5% for P1 and P4 respectively in 2013 (Fig 7).

## Discussion

The olive tree has several means to balance and control fruit load while considering resource availability as reflected by tree nutritional status [4, 6, 13]. While flowering induction does not seem to be controlled by the total carbohydrate availability [25], flower gender and quality parameters are closely related to resource availability (N and carbohydrate) [29, 31] and fruit set is determined accordingly [5, 28]. In the current study, elevated P nutritional status was shown generally to enhance female expression of flower quality and final fruit set. We previously speculated that the positive effect of P nutrition on perfect flower formation and fruit set is related to carbohydrate availability and transport dynamics [32]. However, the current study indicates that the positive effect of P is independent of the total carbohydrate availability to the reproductive organs (excluding pollen).

Low flower load is understood to be associated with higher flower quality [4, 51] and consequently, higher rate of fruit set [5, 6, 52]. In contrast, low P availability impairs both flower intensity and fruit set as shown in this (Table 1) and a previous study [32]. The diminishment of both flower intensity and fruit set at low P status indicates the centrality of P for reproductive success. Flower quality parameters including the portion of perfect flowers, inflorescence and pistil biomass, and, to a certain degree, pistil persistence and fruit set, along with flower production, were all positively associated with P nutritional status. Conversely, starch and SCH concentrations were not enhanced by P. In a previous study the positive effect of P nutritional level on the ratio of perfect flowers was demonstrated to be more pronounced in highly inductive conditions [32], probably as a result of greater competition for resources. We therefore conclude that the positive effect of P found on flower quality might have been more pronounced if flowering intensity was higher.

Pistil biomass is considered an important trait, positively related to sink strength [13] and hence, large pistils have increased probability for successful fruit set. In the two studied seasons, increased P availability was accompanied by a continuous increase in pistil biomass in both apical and basal positions (Fig 5). The effect of P enhancement was more pronounced on the basal, less nurtured positions. In a study by Cuevas and Polito [12], male flowers tended to develop at secondary, less competitive, positions. Here, we show that also within the population of perfect flowers, ovary biomass is related to its position within the inflorescence, and bigger pistils are likely to occur in the apex. This intra-inflorescence distribution pattern indicates a second level of resource allocation to positions with better chances of fertilization. High P nutritional level inhibits the competition among perfect flowers within the inflorescence. For example, in 2012 the pistils in basal positions had 34, 24, 15 and 11% lower biomass compared to those in the apex for P1–P4, respectively.

Substantial flower and fruitlet abscission occurs closely after FB [17]. The later abscission is generally related to competition among fertilized ovaries [3]. In the current study, abscission dynamics were very similar to the pattern reported for ‘Manzanillo’ by [2]. At low P levels reduced pistil persistence was found while high P levels were associated with greater endurance, especially of the male flowers (Fig 6). This is likely to have no significant implication on productivity since the effective pollination period of the olive is probably no more than three days [53]. The different abscission dynamics might rather indicate greater sink strength and better flower quality of high P trees.

Successful fertilization requires viable pollen from male reproductive organs, capable of germinating, growing and fertilizing one of the ovules. This process is fundamentally superior in the case of cross-pollination [18, 20], hence, pollen quantity and viability is important for productivity of neighboring cultivars. Contrary to the features of female flower quality, pollen viability was considerably and consistently reduced as P levels increased (Fig 7). A study conducted on *Lilium* revealed close association between starch levels and pollen development [54]. Supposedly, for P deficient trees, the combination of the higher starch reserves found in the inflorescence with fewer anthers to compete for carbohydrates might have contributed to the elevated pollen viability of these flowers. If so, P deficiency impairs the female organs while indirectly improving male function. There was relatively large difference between the two evaluation methods of pollen viability rate and variability between season for given method (Fig 7). In general, both methods indicated similar trends, i.e. diminishing viability with P level. The Alexander reagent tends to over-estimate viability [55] while the FDA method was shown to be closely related to direct germination test and thus considered more reliable for olive pollen [56]. The FDA values (30–9.5%) are well in agreement with the range found for six Croatian varieties over six years (32–2%) by direct germination tests [7].

Phosphorus deficiency is known to negatively affect photosynthesis processes [57], probably due to its central role in trios phosphate transport from the stroma [34, 58]. Hence, under P deficiency starch and SCH are expected to accumulate in the source organ (leaves) and diminish in the sink organs (flowers and fruitlets) [59]. The present study’s results are in agreement with the reported inhibitory effect of P deficiency on *A*_*n*_ and enhanced starch accumulation in the leaves (Figs 2 and 3), but no significant effect of P on the measured SCH was found. The combined processes is expected to lead to substantial decrease in assimilate availability to sink reproductive organs. Such effect may be most pronounced during the period of flowering and fruit set when carbohydrate demand is highest. Starch reserves were previously demonstrated be correlated to flower viability of several fruit trees including apricot [21], avocado [23, 24], and citrus [60]. In olives, Reale et al. [27] showed that ovary and ovule starch levels were closely related to perfect flower development. Rapoport et al. [61] reported reduced starch grain presence in ovaries and ovules following drought stress which subsequently resulted in reduced fruit set. The above information, in combination with the positive associations between higher leaf/flower ratio and girdling to fruit set [28, 31] indicates that increased carbohydrate level positively effects flower quality and fruit set.

In the current study, assimilation inhibition of P deficient trees was evident during the period of fruit set. The decrease in *A*_*n*_ was accompanied by a minor decrease of *g*_*s*_. The slightly lower *g*_*s*_ can only partially explain the impaired assimilation *A*_*n*_ is relatively insensitive to changes in *g*_*s*_ at high values due to the asymptotic nature of its response curve [62]. Therefore, inhibition in *A*_*n*_ at the lowest P level is likely to be mainly due to slower trios-phosphate export and consequently, feedback inhibition. Starch accumulated in the leaves and inflorescences of P deficient trees (Fig 3), while SCH concentration was not modified by P. These results lead to the conclusion that, in spite of the impaired *A*_*n*_, carbohydrate availability did not limit reproductive processes of P deficient trees. While contradicting our initial hypothesis, comparable analogs for this conclusion can be found in the literature for other species. For example, in P deficient rice plants, *A*_*n*_ and root growth were simultaneously diminished. By manipulation of light intensity Wissuwa et al. [63] demonstrated that root growth inhibition was not a cause of assimilate limitation (source limitation) but was directly related to P deficiency (sink limitation). Similarly, we suggest that in P deficient olive, carbohydrate supply does not limit productivity but rather that the development of reproductive organs is restricted by P availability. Reduced viability of flowers with decreased P availability is further revealed by the abscission dynamics of the flowers (Fig 6). Flowers from trees with low P tended to abscise earlier. Fast flower and fruitlet abscission indicate lower sink strength of low P flowers rather than limited carbohydrate supply, as previously demonstrated for citrus [60]. In the current study, total available starch and SCH did not seem to play a major role in olive reproductive processes. In spite of this, P and carbohydrates may be involved by means of signaling and alternation of specific sugars or phosphorylated sugars. Specific carbohydrate forms such as phosphorylated hexose sugars have been shown to mediate metabolic, enzymatic or genetic possesses [64]. For example, sucrose and malate were suggested to serve as signals controlling stomatal movement [65, 66]. While these indicate a potential involvement of specific carbohydrates affected by P level, only minor SCH and SCH phosphorylation were determined.

Several studies investigated seasonal SCH dynamic of various tissues of the olive tree (i.e.) [26, 67, 68]. These studies, supported by Stutte and Martin [25], indicated lack of association between carbohydrate reserve level and flower formation. De la Rosa et al. [69] demonstrated starch accumulation in buds at the onset of olive differentiation. Starch granule formation is likely vital for proper pistil formation and function [27]. In the current study, starch was highest close to initiation and decreased during inflorescence development while starch content per inflorescence increased (Fig 3). The initial high concentration derives from starch accumulation during the winter [69]. The SCH concentration showed dynamic behavior; at the first developmental phase, SCH tended to decrease with time, probably an indication of dependency of the young inflorescence on SCH and starch reserves. Subsequently, during the rapid development phase of inflorescence, SCH increased to its highest values. High carbohydrate values may serve the developing flower in proper pollen development [54] and support future fertilization and fruitlet persistence [23, 24]. The pronounced decrease in biomass, starch and SCH during and subsequent to FB is likely related to release of pollen grains and enhanced metabolic processes. Decrease in inflorescence dry mass a few days before anthesis was also reported by Kitsaki et al. [70].

To conclude, P nutritional level was shown to be positively associated with flower quality parameters, mainly due to lower occurrence of pistil abortion and increase in ‘perfect’ pistils and inflorescence biomass. These traits are likely to enhance the sink strength of flowers which is positively related to pistil and fruitlet persistence and final fruit set. The positive impact of P nutrition was neither related to total carbohydrate reserves nor carbohydrate transport to the developing inflorescence. Despite having inferior *A*_*n*_, low P trees accumulated carbohydrates in the inflorescence to levels which are comparable or higher than trees exposed to high levels of P. Under P deficiency, the combination of low flower production, inferior flower quality, low fruit set and high carbohydrate reserves implies that P shortage directly limits reproductive processes, from flower initiation to fruit set. The higher carbohydrate reserves found in low P inflorescences might have contributed to the significant increase in pollen viability.
